# Improving Procedural Skills, Teamwork and Confidence in just One Day: A NICU Fellow Boot Camp

**DOI:** 10.15694/mep.2018.0000247.1

**Published:** 2018-11-07

**Authors:** Sadie Williams, Beth Ann Johnson, Gary Geis

**Affiliations:** 1 The Perinatal Institute; 2 The Perinatal Institute; 3 Department of Emergency Medicine and Simulation

**Keywords:** orientation, simulation, fellowship, bootcamp, task training

## Abstract

This article was migrated. The article was marked as recommended.

**Objective:** To develop a compact, simulation-based orientation session aimed at improving confidence and teamwork amongst new trainees.

**Methods:** Two cohorts of five fellows participated in the one-day boot camp. Confidence in team leading and neonatal procedures was assessed using a pre and post survey administered on the day of boot camp as well as a delayed post-test after 6 months. Teamwork and communication were assessed using the TEAM scale.

**Results:** We found that fellows confidence was significantly improved in 5 out of 6 categories (team leader in code (p < 0.001), team leader in delivery room (p < 0.001), decision making (p < 0.001), intubation (p < 0.001), chest tube placement (p < 0.001) and umbilical catheter placement (p = 0.09)). There was an overall improvement in teamwork, team leadership and communication throughout the day (p < 0.001). There was no significant change in self-reported confidence scores in all categories 6 months following boot camp.

**Conclusions:** We demonstrated a significant improvement in confidence in first year neonatal-perinatal medicine fellows following a one-day simulation-based boot camp. The format for this boot camp could be emulated in institutions across the country to improve the confidence and skills of incoming fellows.

## Introduction

The first month of fellowship training can be an overwhelming transition. Unfamiliarity with a new medical institution, uncertainty regarding complex medical diagnoses and inexperience with procedural and communication skills can contribute to the challenge. ( Nishisaki *et al.*, 2009; Young *et al.*, 2011; Cohen *et al.*, 2013; Wen *et al.*, 2015) In the neonatal intensive care unit (NICU), incoming fellows have had increasingly limited experiences due to work hour restrictions and may be further limited by the level of acuity at their previous institution. Beyond this, the role of a fellow is different than that of a resident, with increased clinical and leadership responsibilities, further adding to the stress. An orientation experience to onboard fellows to the institution and the position may help smooth this transition and reduce their stress.

Across the country, neonatal fellowship programs do not have a standardized process for orientation leaving individual fellowships to develop an onboarding process. To help fill this need, an orientation curriculum, based on specific institutional needs assessments should be developed. A structured “boot camp” during the onboarding process could help bridge this gap while improving confidence and performance. Boot camps utilize various modes of teaching, including: simulation-based exercises, focused procedural skills (task training) sessions and resuscitation leadership opportunities in an arena similar to the patient care environment. ( Blackmore *et al.*, 2014)

High fidelity simulation-based boot camps lasting days to months are emerging throughout many procedural focused specialties as a way to teach leadership, communication and procedural skills to a group of trainees with various levels of experience and training. This type of simulation training has the potential to decrease medical errors secondary to lack of practice. ( Nishisaki *et al.*, 2009; Bismuth *et al.*, 2012; Cohen *et al.*, 2013; Ataya *et al.*, 2015; Heskin *et al.*, 2015) In addition, simulation training provides a unique opportunity for training that will not cause patient harm yet has been shown to induce stress and increase cortisol levels in neonatal fellows, similar to a real-life emergency. ( Finan *et al.*, 2012)  Despite these potential advantages, a NICU fellow boot camp has not yet been described in the literature. Furthermore, literature describing the possible benefits of a boot camp lasting only one day, in any specialty, is lacking.

The goal of this study was to develop a compact, one-day simulation-based orientation session utilizing didactics, simulation scenarios and debriefing sessions. The sessions were focused on critical neonatal diagnoses and teamwork, and deliberate practice focused on life-saving neonatal procedures. We hypothesized that this type of orientation session would (1) improve first-year fellow confidence in their ability to perform critical procedures and (2) improve first-year fellow teamwork in resuscitative care.

## Methods

### Setting and Subjects

We conducted a prospective study at Cincinnati Children’s Hospital Medical Center (CCHMC) of two cohorts of incoming clinical fellows in 2016 and 2017 with repeated measures before and after a one-day boot camp. Training was delivered in an on-campus, simulation lab that included a debriefing room, two simulation rooms and a control room. Supplies, including different non-invasive ventilation, intubation and chest tube equipment, from three hospitals were used to simulate the environment of all NICUs where fellows work. Simulations were video recorded to allow application of measures of outcome. Participation in boot camp was mandatory for all fellows but participation in the study was optional. This study was approved by our institutional review board prior to enrollment.

### Study Aim and Outcomes

The aim of this study was to develop and implement a simulation and deliberate practice-based orientation session or boot camp for new fellows focusing on teamwork, leadership and procedural skills. In doing this, our primary outcome was to determineif a one day NICU fellow boot camp improved the participant confidence in the ability to perform key procedures (intubation, chest tube placement and UVC placement) and lead resuscitation scenarios. Our secondary outcome was to determineif the boot camp was able to improve teamwork amongst new fellows measured using the Team Emergency Assessment Measure (TEAM) scale. Our tertiary outcome was to determine whether participants maintained confidence six months following completion of boot camp.

### Simulation and Debriefing

A total of five simulation scenarios were developed and piloted for feasibility, conceptual accuracy, and reliability after performing an online needs assessment of current neonatology fellows and faculty. A description of the scenarios is included in Appendix 1. Each fellow was required to act as team leader for one simulated resuscitation. Debriefing occurred following every scenario and focused on the non-technical behaviors (teamwork, leadership and communication skills) as well as cognitive/knowledge objectives (assessment, medical decision making, and reassessment). This was led by a facilitator formally trained in simulation debriefing, a faculty-level neonatologist and education specialist from the Center for Simulation. To augment the cognitive/knowledge objectives of each scenario, a 15 minute didactic segment by a faculty-level neonatologist was included after the debriefing.

### Deliberate Skills Practice

Following three of the core simulation scenarios, there were deliberate practice training sessions focusing on endotracheal intubation, chest tube placement/needle thoracostomy, and umbilical catheter placement. This training was performed under the direction of senior fellows, attending physicians and/or neonatal nurse practitioners. Task trainers and simulators, which were selected based on previous experiences of the educators, are listed in Appendix 1.

### Data Collection and Outcome Measures

Participant confidence was measured using self-reported pre and post-surveys (Appendix 2) with Likert scale style questions using a 1 to 10 scale, administered prior to the first scenario, following completion of all five scenarios and 6 months after boot camp. The post-tests were identical to the pre-test in content. Team-level performance was assessed using the TEAM scale via video review of the first and last scenario. The TEAM scale is a 12-item tool that has been validated in simulation-based training to evaluate the non-technical domains of team leadership (items 1-2), teamwork (items 3-9) and task management (items 10-11). The tool had a high Total Content Validity Index (0.96), internal consistency (0.89) and concurrent validity demonstrated by high correlation of the items with the global rating item. Intra-class correlation was included with inter-rater and retest rating of 0.60 and 0.80 respectively. ( Cooper *et al.*, 2010)This review was performed by an expert in simulation who has been trained in application of this tool.

### Statistical Analysis

A two sided t-test was used to compare the continuous variables on pre and post-tests. For our secondary outcome, the mean differences between the first and last scenario were analyzed using one-way between scenario ANOVA. A two sided t-test was again used to compare the continuous variables between the pre, post and delayed post-tests to compare retention of confidence.

## Results/Analysis

### Study population demographics

We enrolled ten incoming neonatology fellows, five in 2016 and five in 2017. All ten fellows completed their three year pediatric residency through an accredited program in the United States. Three of the participants completed an additional year as chief resident. One of the trainees had completed residency at CCHMC prior to enrollment in boot camp. Self-reported previous experiences are listed in Table 1. All trainees were certified by the Neonatal Resuscitation Program (NRP) prior to enrollment in the boot camp.

**Table 1.** Summary of self-reported clinical experiences prior to boot camp

Previous Clinical Opportunity	Percent with previous experience (n = 10)	Median number of experiences
Team Leader in Simulation	100%	n/a
Team Leader in Neonatal Resuscitation	70%	n/a
Team Leader in Pediatric Code	40%	n/a
Intubation	100%	9
Needle Decompression	50%	0.5
Chest Tube	50%	0.5
UVC/UAC	100%	12.5

### Confidence Scores

Confidence scores were significantly improved between pre and post-test assessment in five of the six categories assessed ( Table 2).

**Table 2.** Pre-simulation training mean confidence ratings vs post-simulation training mean confidence ratings

	Pre-Test Mean +/- SD	Post-Test Mean +/- SD	P value
Team Leader in Code	5.0 +/- 2.2	7.8 +/- 1.5	< 0.05
Team Leader in Delivery Room	6.3 +/- 2.1	8.2 +/- 0.9	< 0.05
Make Medical Decisions	6.7 +/- 1.5	7.4 +/- 1.4	< 0.05
Perform Intubation	6.4 +/- 2.7	7.7 +/- 1.8	< 0.05
Place Chest Tube	3.6 +/- 2.2	6.9 +/- 2.1	< 0.05
Place Umbilical Catheter	7.1 +/- 2.2	8.2 +/- 1.6	0.09

### Team Level Performance

The first-year fellows’ non-technical performance was analyzed using the TEAM scale, comparing performance in scenarios 1 and 5. For line items 1-11 (scale range 0-4 for each item) performance improved in scenario 5 for all line items, with exception of line 2, which remained the same between scenario 1 and 5 in year 2. For the overall global assessment rating (scale range 1-10), the reported scores were 3 and 4 for the first scenario in years 1 and 2 respectively and 8 in the fifth scenario in year 1 and 2. One way ANOVA was performed for the combined 2 year TEAM scale analysis and revealed a statistically significant difference between scenario 1 and scenario 5 with a p value of

### Maintenance of Confidence at Six Months

Over the first 6 months of training, all ten fellows reported acting as a team leader, performing intubations and umbilical catheter placement. Nine of the ten fellows had the opportunity to place a chest tube. There was no statistical difference between the post-test confidence ratings and the delayed post-test confidence ratings. ( Table 3).

**Table 3.** Post simulation training mean confidence ratings vs delayed post-simulation training mean confidence ratings

	Mean Clinical Opportunities +/- SD	Post-Test Mean +/- SD	Delayed Post-Test Mean +/- SD	P value
Team Leader in Code	8.5 +/-6.0	7.8 +/- 1.5	7.4 +/- 1.3	0.56
Team Leader in Delivery Room	23.8 +/- 31.7	8.2 +/- 0.9	8.6 +/- 1.1	0.31
Make Medical Decisions	N/A	7.4 +/- 1.4	7.9 +/- 1.1	0.69
Perform Intubation	14.4 +/- 8.6	7.7 +/- 1.8	8.5 +/- 1.3	0.16
Place Chest Tube	3.4 +/- 2.2	6.9 +/- 2.1	7.3 +/- 2.2	0.72
Place Umbilical Catheter	8.4 +/- 5.3	8.2 +/- 1.6	8.8 +/- 0.9	0.30

## Discussion

Following the implementation of boot camp, we saw a significant improvement in the mean self-reported confidence scores for fellows in making medical decisions, acting as a team leader during a code and neonatal resuscitation, chest tube insertion and intubation. Our findings support the use of high fidelity simulation orientation sessions to improve participant confidence in the ability to perform key procedures and lead resuscitation scenarios and highlights the possibility of limiting the sessions to one day.

As a measure of teamwork and communication, the TEAM scale was employed to evaluate the changes in non-technical behavior between the first and last scenario. Fellows demonstrated an overall improvement in teamwork, team leadership and communication throughout the day. This parallels the participants’ improved confidence in their ability to perform as team leaders in resuscitation scenarios. It is not surprising that non-technical behaviors improved, as these behaviors were the focus of debriefing after scenario 1 and were part of the discussion in debriefings after scenarios 2-4. However, this improvement speaks to the effectiveness of simulation-based training for non-technical skills when emphasized during simulations and debriefings. ( Burton *et al.*, 2011; Patterson *et al.*, 2013)

We found no significant change in confidence scores six months following the training session compared to scores at completion of the training session. This could be interpreted as a maintenance of confidence throughout the first six months of fellowship. However, this may also indicate that new fellows gain confidence in the session that would ordinarily be obtained in the first six months of fellowship. This would demonstrate that the boot camp allows fellows to start fellowship with a level of confidence that is typically developed several months into fellowship and that they maintain this confidence as they continue to hone their skills.

The biggest limitation to this study is the small sample size. The sample included only ten neonatal-perinatal fellows. Fortunately, each of the fellows participating in boot camp came with a different background and training experience, allowing us to assess the effect of the boot camp on a small but diverse sample. Second, this boot camp was performed with incoming fellows at only one institution, limiting generalization of our findings to additional institutions. In the future we hope to dampen these limitations by continuing to offer this boot camp each July, prospectively collecting data on the pre and post confidence ratings of new fellows here and at other institutions initiating similar boot camp type simulation sessions increasing our sample size and offering better generalizability to findings at locations different from ours.

As shown by self-report at 6 months, the fellows had to team lead in the delivery room and at codes, as well as perform all three procedures clinically.  While improving confidence in trainees is valuable, it is important to determine if this type of training has an impact on clinical performance. Previous studies have demonstrated an improvement in technical skill, teamwork, and communication in simulated patient scenarios as well as improvement in time to recognition of disease process and escalation of patient care following implementation of longitudinal staff simulation training programs. ( Bang *et al.*, 2016; Murphy *et al.*, 2016; Taira *et al.*, 2016; Rabago *et al.*, 2017; Theilen *et al.*, 2017)However, research demonstrating clear impacts on patient outcomes is lacking. ( Neylan *et al.*, 2017)Future studies should aim to evaluate the impact of this type of training on not only resident and fellow self- perceived confidence of skill but also impact on performance and outcomes in the clinical realm.

## Conclusion

In a time when incoming fellows are potentially arriving with less overall procedural, leadership and resuscitation skill experience, we demonstrated a significant improvement in self-perceived skill confidence, teamwork, and team leadership in first year neonatal-perinatal medicine fellows following a one-day high fidelity simulation-based boot camp. The format for this boot camp is feasible and could be emulated in institutions across the country as a first step to improve the confidence and skill set of incoming fellows.

## Take Home Messages

• A one-day simulation-based boot camp experience is a feasible and efficient strategy to introduce teamwork and procedural skills to incoming trainees.
• This one-day experience improves teamwork, team leadership and communication measured over the course of the day, while also increasing confidence in team leadership prior to the onset of clinical care.
• This one-day experience increases confidence in performing critical procedural skills prior to the onset of clinical care.
• Confidence levels reported after the boot camp were mirror confidence levels reported by trainees six months into fellowship.
• This one day boot camp would be easily reproduced in a variety of training centers.

## Notes On Contributors

Sadie Williams recently completed fellowship in neonatal-perinatal medicine at Cincinnati Children’s Hospital Medical Center where she worked to improve the education of trainees within the department of neonatology. As a member of the Center for Neonatal Care at Florida Hospital, she plans to continue working to improve the education of trainees in the NICU.

Beth Ann Johnson is an Assistant Professor within the Perinatal Institute at Cincinnati Children’s Medical Center. Her primary focus is on improving the clinical care of neonates through education of medical residents, fellows and NICU staff.

Gary Geis, an Associate Professor in the Division of Emergency Medicine and Medical Director of the Center for Simulation and Research, has 18 years of experience in medical simulation including institutional recognition as an educator and federal funding from AHRQ to implement and evaluate simulation-based training for medical trainees.

## Appendices

**Appendix 1.** Description of simulation sessions, skills required during the session and associated deliberate skills practice sessions following the simulation

Session	Description	Procedures During Resuscitation	Deliberate Skills Practice and Materials Used
1	**Hydrops Fetalis – Megacode ** Infant born via stat C-section to previously healthy 31 year old G2P1 female presenting at 30+2 weeks with newly diagnosed severe hydrops fetalis of unknown origin, preterm labor and decreased fetal movement. Ultrasound prior to delivery revealed generalized edema as well as bilateral pleural effusions and decreased heart tones. On arrival, infant with heart rate of 10 and absent respiratory effort. Exam significant for generalized edema, muffled heart sounds and decreased breath sounds as well as distended abdomen. *Scenario focus is on teamwork and communication. Infant is unable to be resuscitated.	-Bag Mask Ventilation -Chest Compressions -Intubation-UVC Placement -Needle Decompression -Chest Tube Placement Optional: -Paracentesis -Pericardiocentesis	Main Skills Focus: - Teamwork - Communication Task Trainers Used: Laerdal SimBaby
2	**Obstructed Endotracheal Tube (ETT)** Infant is a 7 week old former 23 week male who was originally transferred at 2 weeks of life with necrotizing enterocolitis with perforation s/p treatment. He is now working on increasing nasogastric feeds. He remains on the ventilator (Pip 28, Peep 8, PS 10, Rate 35) and has been having increasing secretions and need for higher pressures over the last 3 days. A code is called. On arrival, ETT has been disconnected from ventilator and bag is attached to ETT to provide manual ventilation. The bedside RN states that his sats progressively dropped over 2 minutes despite increasing oxygen to 100%, heart rates then began to fall and neither saturations nor heart rate has responded to hand ventilation. On exam: HR 67, SpO2 28%, RR 4, BP 79/43 (MAP 46) with no spontaneous respiration of movement and extremely decreased but equal aeration bilaterally without chest rise	- Bag Mask Ventilation - Rapid Sequence Intubation (with pre-medication)	Main Skills Focus: - Laryngoscopy - Intubation Task trainers Used: Laerdal SimBabyLaerdal Premature Anne (2) Simulab TraumaChild
3	**Pneumothorax** Infant is a 1 day old male born at 33 weeks EGA via precipitous vaginal delivery to a 32 year old G1P0 with preterm labor. He required only CPAP in the delivery room, and he was brought back to the NICU where he remained on CPAP +5, 30% oxygen for several hours. Over the last hour his oxygen requirement has been increasing and he is now at 70% oxygen with continued desaturations. In the last 10 minutes, oxygen has been increased to 100%. On exam: HR 157, RR 92, SpO2 87%, BP 58/33 (40). He has decreased breath sounds on the right compared to left.	-Needle Decompression -Chest Tube Thoracostomy	Main Skills Focus: - Needle Decompression - Chest Tube Thoracostomy Task trainers used:Laerdal SimBabySimulab TraumaChild
4	**Placental Abruption ** Called to a stat C-section for mom at 39 weeks 1 day with history of classical incision with prior delivery. Mom presented to triage with bleeding and non-reassuring fetal surveillance. Remainder of pregnancy has been normal with negative prenatal labs. Estimated fetal weight on ultrasound 2 days ago was 3000 grams. Following delivery, there is no spontaneous respiratory effort noted, heart rate can be palpated at 5 seconds	-Bag Mask Ventilation -Chest Compressions -Intubation -UVC Placement	Skills Focus: - UVC Placement Task trainers Used: Laerdal SimBabyLaerdal Premature Anne (2)
5	**Unstable Supraventricular Tachycardia (SVT)** You are called by a community pediatrician regarding a former 29 week infant who is now 42 weeks corrected. His hospital course was uncomplicated with exception of intermittent SVT, treated with propranolol. His episodes typically self-resolved prior to intervention while in the NICU. The infant presented to the office today for follow up and was found to have heart rate of 220 but was been hemodynamically stable. You instruct the pediatrician to have EMS bring the baby from the clinic across the street for admission to the NICU. EMS places an IV in the foot in route but they do not administer medications. On arrival: T 39, HR 220, RR 79, MAP 45, SpO2 95% on room air. Infant is awake, all pulses are palpable but extremities are mottled. Capillary refill initially Throughout the scenario, despite vagal maneuvers and administration of Adenosine, infant remains in SVT	-Vagal Maneuvers -Bag Mask Ventilation -Intubation -Chest Compressions -Synchronized Cardioversion	Main Skills Focus: -Defibrillator use Task Trainers Used: Laerdal SimBaby

### Appendix 2.

Bootcamp Simulation Pre- and Post-test:

On a scale of 0-10, with 0 being not at all comfortable or confident and 10 being completely comfortable and confident, how comfortable/confident are you:

In your ability to be the team leader/run a code?

In your ability to be the team leader/run a delivery room resuscitation?

In your ability to effectively make medical decisions?

In your ability to effectively deliver bad news to parents/family?

In your ability to perform intubations?

In your ability to place a chest tube?

In your ability to place a UAC or UVC?
