# Supplementary material for: Improving Procedural Skills, Teamwork and Confidence in just One Day: A NICU Fellow Boot Camp
Source: MedEdPublish (2016). 2018 Nov 7;7:247. [Version 1] doi: 10.15694/mep.2018.0000247.1 (PMC10711953; doi:10.15694/mep.2018.0000247.1)
Supplement: Peer review history

# Reviewer report: Reviewer response for version 1

**DOI:** 10.21956/mep.19700.r29461

**Published:** 2018-12-11

**Authors:** Michael SH Wan

**Affiliations:** 1 University of Notre Dame

This review has been migrated. The reviewer awarded 4 stars out of 5 Thanks for a well written paper describing how scenario based simulation boot camp can improve confidence in various emergency procedural skills as well as communication and team building.The description of the day was detailed and the analysis sound. The 6-month retention was encouraging showing sustained effect of the camp.Would be good if the selection of the case scenarios was described in more detail and if the content of the scenarios could be shared (via a link) for other institutions to reference to (if copyright permits).Extension to other emergency scenarios and other disciplines would be very useful.

Reviewer Expertise:

NA

No decision status is available

# Reviewer report: Reviewer response for version 1

**DOI:** 10.21956/mep.19700.r29460

**Published:** 2018-11-25

**Authors:** Stuart Marshall

**Affiliations:** 1 Monash University

This review has been migrated. The reviewer awarded 4 stars out of 5 This interesting paper suggests that a ‘boot camp’ for Neonatal ICU fellows can improve team performance and confidence. Confidence was sustained after a six month duration. The teaching component included clinical skills and non-technical skills for five scenarios.Although not a new idea, this is well-written example of how such a program should be developed and assessed and the authors should be congratulated on a successful program.I’d be interested to hear more about how the five scenarios were determined. The authors say a ‘needs analysis’ was undertaken of fellows and faculty but it’s unclear whether they were identifying the high risk scenarios, where previous gaps had been noted, or something else.It’s also not currently clear if the scenarios were randomised. If they were presented in the same order (and the last scenario was easier than the first) it could introduce a systematic bias into the assessment.The TEAM scores are not fully reported. It would be useful to see those statistical tests. My concern is that the ANOVA test might not be appropriate due to non-normality of data arising from the granularity of the scale. Nevertheless, the results superficially look robust.I’d be interested in seeing a trial with a control of ‘traditional’ teaching compared to the boot camp method.

Reviewer Expertise:

NA

No decision status is available

# Reviewer report: Reviewer response for version 1

**DOI:** 10.21956/mep.19700.r29459

**Published:** 2018-11-21

**Authors:** Ken Masters

**Affiliations:** 1 Sultan Qaboos University

This review has been migrated. The reviewer awarded 4 stars out of 5 A very well written and detailed paper, measuring and discussing the impact of a procedure and teamwork boot camp on both perceptions of, and actual, abilities. Although the authors have correctly noted the limitations of one institution and small sample size, the result are certainly encouraging, and indicate that this process could be repeated, both by the authors and by other institutions. The six-month follow-up is a useful rounding to the study.Although external to the paper, it would be of value (copyright permitting) if the authors could put links to their teaching and other materials, so that other institutions may implement a similar boot camp.Some small issues:• Early in the Introduction, the authors write “Across the country, neonatal…” with no indication of which country. Based on the authors’ affiliations and the later mention of the study location, one senses that it is the USA, but this needs to be stated explicitly.• A few minor punctuation and spacing errors, especially lack of commas. In most cases, it is just an irritation; in some cases, it affects meaning. An example is the sentence containing “patient harm yet has been shown” which definitely needs a comma before the yet. (As it stands, it means that it will not yet cause patient harm). Also “determineif” and others.

Reviewer Expertise:

NA

No decision status is available

# Reviewer report: Reviewer response for version 1

**DOI:** 10.21956/mep.19700.r29458

**Published:** 2018-11-17

**Authors:** Megan Anakin

**Affiliations:** 1 University of Otago

This review has been migrated. The reviewer awarded 4 stars out of 5 Overall, the authors provide a well-written description of their study and situate it in relation to current practice needs and literature. The introduction clearly outlines the background of, and the need for, this study. The goal of the study was develop a simulation ‘boot camp’ intervention to show improved teamwork, leadership and procedural skills. The methods provide a succinct description of the study’s context and components supported with more information about the scenarios and survey instruments placed in appendices. The results are presented in well organised tables supported with statements that help the reader interpret them. As expected, participants in the study showed improvement after the ‘boot camp’ and these gains were maintained after six months. The authors report several limitations of their study. Another important limitation of the study was its focus on the intervention only. The study was limited to participants in the ‘boot camp’; there was comparison group. Despite this limitation, the authors situate their findings appropriately in relation to recent studies with a focus on improving technical skills, teamwork, and communication. To enhance this article, the authors might consider providing readers with more signposting in the introduction that this article reports on a ‘proof of concept’ or feasibility study. If this signposting were added then the article would emphasise the study’s strengths to a greater degree because educators who have worked to develop engaging simulation scenarios will appreciate that this endeavour is challenging. To further support the claims made in the discussion, the authors might consider conducting a future study that compares their ‘traditional’ teaching of resuscitation with the simulation ‘boot camp’ technique.This article will be of interest to educators with an interest in simulation.

Reviewer Expertise:

NA

No decision status is available
